# “It’s Not the Same”: A Comparison of the Psychological Needs Satisfied by Musical Group Activities in Face to Face and Virtual Modes

**DOI:** 10.3389/fpsyg.2021.646292

**Published:** 2021-06-02

**Authors:** Grace Draper, Genevieve A. Dingle

**Affiliations:** UQ Music, Dance and Health Research Group, School of Psychology, The University of Queensland, St Lucia, QLD, Australia

**Keywords:** group singing, dance, instrumental groups, social identity approach, virtual, psychological need satisfaction

## Abstract

According to the social identity approach to health, group memberships influence people’s mental health to the extent that they identify with their group. Emerging evidence suggests that music groups, such as choirs, enhance mental health *via* group identification and the satisfaction of various psychological needs; however, more research is required to understand these processes in other types of music groups. Furthermore, the coronavirus disease 2019 (COVID-19) social distancing restrictions in 2020 prevented music groups from meeting face to face (F2F). Some music groups adapted virtually, but the rate of adaptation of various music activities is unknown, as is the impact of such adaptations on participants’ group identification, psychological need satisfaction, and mental health. We explored these questions using a cross-sectional survey with 257 participants (*M*_*age*_ = 46 years, 78% female) of singing (*n* = 172), instrumental (*n* = 48), and dance groups (*n* = 37). Participants rated group identification and psychological needs satisfaction retrospectively for their music group in F2F mode and then for the group in adapted mode, along with mental health 12-item short form health survey (SF-12). Results showed that instrumental groups (60%) were less commonly adapted to virtual mode than singing (83%) and dance (86%) groups. Group identification and average psychological needs satisfaction (*M* = 4.04 and 3.50 out of 5) scores were significantly lower for groups in virtual mode than in F2F mode (*M* = 4.53 and 4.14, respectively). Psychological needs satisfaction did not mediate the relationship between group identification and SF-12 mental health. Despite this, values on group identification and psychological need satisfaction remained high, which suggests that virtual music groups may be beneficial during the COVID-19 pandemic and in contexts where F2F groups are less accessible.

## Introduction

This study addresses the broad question of whether–considering coronavirus disease 2019 (COVID-19) social distancing restrictions–it is possible for musical group activities to be successfully adapted to virtual forms while maintaining their psychosocial benefits for participants. Following on from initiatives, such as Eric Whitacre’s virtual choir that launched in 2010, advancements in technology have offered new ways for musical groups to function (Whitacre, 2018). Widespread use of videoconferencing platforms has enabled the development of virtual musical experiences, such as virtual choirs and orchestras, live streaming of performances, and online interactive music making. While this had previously been considered a niche option, there has been a rapid rise in virtual adaptations of musical groups during the COVID-19 pandemic because restrictions on social gatherings have curtailed face to face (F2F) musical group rehearsals and performances. International examples seen on social media include Italians singing with their neighbors across their balconies, dancers from New York City Ballet posting videos of their dancing at home and in the outdoors during lockdown, and an Australian public singing project called “Pub Choir” becoming a virtual “Couch Choir” (Hinchliffe, 2020; Kearney, 2020; Stahl, 2020). Relative to F2F, it is unclear whether participants in these virtual music groups retain their group identification and psychological need satisfaction from the group.

We can look to social and organizational psychology for some evidence, such as the finding that work groups transitioning from F2F to virtual platforms experience a reduction in group identification following the reconfiguration (McGrath et al., 1993; Bouas and Arrow, 1995). This is also supported by studies investigating work groups undergoing restructures (e.g., disbanding groups and forming new ones). Members who identified strongly with their work team were more likely to resist restructure changes and experience lower identification with the adapted group (Jetten et al., 2002). This reduction in identification is likely because the new work team is evaluated against the old preferred one, which leads to members having difficulty abandoning the old identity in favor of the new one (Knippenberg et al., 2002; Ullrich et al., 2005).

### Group Identities, Health, and Wellbeing

According to the social identity approach to health, group memberships influence people’s health and wellbeing to the extent that they identify with their groups (Jetten et al., 2011; Haslam et al., 2018). This approach draws upon two well established social psychological theories of intergroup relations. The first is social identity theory (Tajfel and Turner, 1979), which refers to the idea that individuals develop a sense of self from their membership in various social groups (such as family, friendship groups, occupational group, cultural group, and nationality). That is, individuals define themselves not only based on their unique characteristics (personal identity) but also based on the similarities they share with others in their groups and communities (social identities). The second theory, self-categorization theory, builds on this by suggesting that an individual’s response to an event depends on how they define themselves in that context (Turner et al., 1987). For example, people in emergency accommodation who self-categorize as “homeless” show poorer mood and wellbeing than those who reject this category label, regardless of how long they have been in the service (Walter et al., 2015). Since the 1970s, the social identity approach has helped to understand intergroup relations in social, political, and occupational contexts. In the past decade, this line of research has been extended to include health problems and contexts (Jetten et al., 2011, 2014; Haslam et al., 2018).

There is growing evidence that belonging to social groups can influence health and wellbeing in both positive and negative ways, depending on the norms, attitudes, and values the groups hold in relation to health behaviors (Cruwys et al., 2013; Dingle et al., 2015; Miller et al., 2015; Muldoon et al., 2019). For instance, an individual who is trying to maintain a healthy weight may have one group of friends who are supportive and tend to eat healthy meals and a second group of friends who are supportive but who tend to engage in sedentary behavior and overeating. The norms and social influence of this second group may therefore have a negative effect on the health of the individual. Research indicates that it is not so much having contact with groups that influences health, but whether individuals identify with and feel socially connected to others who share their group membership (Jetten et al., 2011; Sani et al., 2012). Indeed, a meta-analysis by Steffens et al. (2019) showed that group interventions designed to promote group identification among participants had a moderate to strong positive effect on physical and mental health outcomes across populations and settings and were more effective if they successfully built intervention group identification. The link between group identification and health outcomes has been found in formal therapy groups, such as cognitive behavior therapy (Cruwys et al., 2020), therapeutic communities for alcohol and other drug treatment (Dingle et al., 2015), group programs for weight management in obesity (Khan et al., 2020), and peer support groups, such as Alcoholics Anonymous (AA) (Taylor et al., 2020).

### Arts-Based Groups, Health, and Wellbeing

Interestingly, however, this effect is not confined to formal therapy groups. Benefits of groups for mental health and wellbeing have also been found in arts-based groups (e.g., Dingle et al., 2021). Evidence that arts-based groups (e.g., painting, creative writing, music making) can improve physical and mental health has been increasingly recognized by health care professionals, governments, and the World Health Organization (All-Party Parliamentary Group on Arts, Health and Wellbeing., 2017; Mansfield et al., 2018; Fancourt and Finn, 2019). Among these arts-based groups, music groups have perhaps been studied the most widely. These include instrumental groups, such as bands and orchestras, singing groups, such as acapella ensembles and choirs, and dance groups, such as ballet and tango. For instance, group singing has been shown to enhance cognitive health in older adults (Särkämö et al., 2014; Dingle et al., 2020), lung health in people with chronic obstructive pulmonary disease (McNaughton et al., 2016), social participation in people recovering from a stroke (Tarrant et al., 2018), quality of life in people with Parkinson’s disease (Irons et al., 2020), improved vitality and mood in people with cancers (Reagon et al., 2017), and improved wellbeing among people experiencing anxiety and depression (Fancourt and Perkins, 2018; Williams et al., 2018). In contrast, the health benefits of instrumental and dance groups are a relatively under-developed area of research. A recent systematic review suggested that singing, instrumental, and dance groups are all effective means for maintaining and promoting wellbeing (Sheppard and Broughton, 2020). Moreoever, Lonsdale and Day (2020) reported no difference between choirs and instrumental groups on nine different wellbeing measures. Taken together, these studies indicate that a range of music activity groups may produce health and wellbeing benefits. However, more research is needed to understand *how* various music groups meet specific psychological needs of participants, and whether different psychological needs are satisfied by different music activities.

### Group Identities Satisfy Psychological Needs

In times of stress, group identification produces health benefits because we are open to giving and receiving various forms of support from members of our ingroups (Haslam et al., 2005). In fact, the more social group identities we hold, the better protected we are against the effects of stressful events (Iyer et al., 2009). Beyond support, ingroup memberships can satisfy other psychological needs, such as belonging, meaning, control, and self-esteem (Greenaway et al., 2016). For instance, Livesey et al. (2012) asked 169 choral singers to reflect on how their choirs contributed to their quality of life, wellbeing, and health. They found that singers’ perception of belonging to a group gave them a sense of togetherness and support, suggesting that singing in choirs might satisfy individuals’ psychological need for relatedness. Expanding on this, a qualitative study with adults experiencing chronic mental health conditions (25 choir members and 23 creative writing group members) revealed that these arts-based groups met participants’ psychological needs for belonging, support, self-efficacy, purpose, and positive emotions (E. Williams et al., 2020). Another study explored how “Finding Rhythms”–a music-making and recording program with incarcerated adults–met the psychological needs of the participants (Kyprianides and Easterbrook, 2020). Results from 104 inmates in 13 prisons showed that the development of a shared “Finding Rhythms” identity led to positive wellbeing by meeting participants’ psychological needs. However, scores were averaged across items about the psychological needs of social support, self-esteem, control, meaning, competence, relatedness, and autonomy, leaving it unclear whether this music program met some psychological needs to a greater extent than others.

It is possible that different group music activities satisfy specific psychological needs to a greater or lesser extent, although this research is exploratory at this stage. Preliminary findings in this field suggest that instrumental groups satisfy members’ psychological needs of autonomy to a greater extent than singing groups (Stewart and Lonsdale, 2016). This may be due to differences in the nature of music activities. For instance, instrumental music groups tend to be organized hierarchically (e.g., lead, first, second violins), which may explain why they satisfy the need for autonomy to a greater extent. In contrast, choir singing tends to be organized in vocal parts (e.g., soprano, alto, tenor, bass) with a flatter hierarchical structure (i.e., a leader and the choir singers with an occasional soloist) who engage in highly communicative, harmonizing, and emotion-evoking activities together. Features such as these might explain why research has found that singing groups satisfy psychological needs for belonging, relatedness, and support. Dance groups have a range of structures including hierarchical troupes (e.g., ballet), collectives of pairs (e.g., ballroom and tango), and synchronized groups (e.g., hip-hop). Dance rehearsals require motor (rather than vocal) synchronization and may therefore emphasize autonomy and control more so than relatedness and support.

### The Current Study

This study employed an international online survey to explore the psychosocial benefits of group music activities during the first wave of COVID-19 in March to June 2020 from the novel perspectives of people who were missing their groups during the lockdown. Using retrospective reports of their usual F2F meetings, we first explored whether there were any differences between music activity groups in terms of the extent to which participants identified with their music group and to which the different groups satisfied various psychological needs of the participants. These included the need to give and receive social support (Haslam et al., 2005), to share feelings and to provide emotional support to others in the group (Dingle et al., 2017), the needs for self-esteem, control, and meaning (Greenaway et al., 2016), and the needs for competence, relatedness, and autonomy (Kyprianides and Easterbrook, 2020). We then examined the extent to which different music activity groups adapted to virtual forms during social distancing, since anecdotal reports indicate that different music activities have different requirements for technology and equipment at participants’ homes, synchronous vs asynchronous musical activities, audio-visual quality, and so on. For the individuals who were attending a virtual music group, we investigated whether such adaptations impacted upon participants’ sense of group identification and the satisfaction of their psychological needs from the music group. Finally, we examined whether virtual music group participation was related to participants’ mental health during COVID-19, *via* its effects on these psychological needs. This mediated model extends on previous similar models tested in Haslam et al. (2005) and Kyprianides and Easterbrook (2020). Thus, the four questions were:

Q1. Prior to COVID-19 when meeting F2F, how did members’ identification with their groups and satisfaction of specific psychological needs differ between group music activities (singing, instrumental, and dance)?
Q2. During COVID-19, what was the difference in proportions of groups that adapted and did not adapt to virtual forms across the three music group activities?
Q3. Did people participating in the adapted groups feel similar levels of group identification and, in turn, psychological need satisfaction with their adapted vs. F2F music group?
Q4. Was there any indication that the relationship between participants’ virtual music group identification (regardless of group type) and mental health status was mediated by the extent to which psychological needs were met by the virtual music group?

## Materials and Methods

This study forms part of the “Missing My Groups during COVID-19” collaborative study between researchers at the University of Queensland (UQ) and Goldsmiths University of London. Two versions of the online survey were developed, with minor differences between them^1^. The surveys were distributed internationally in May 2020 when most countries were in the first wave of the COVID-19 pandemic. Using a cross-sectional design, quantitative and qualitative data were collected. The survey was open to participants missing all group activities but only data pertaining to instrumental, singing, and dance groups are reported in this paper.

### Participants

Participants were eligible to participate if they confirmed that they could understand English and were involved in a group activity before the onset of social distancing restrictions. There was no exclusion criterion for the expertise of the group activity (i.e., amateur to professional). A total of 647 participants were recruited for the study, of whom 257 participants belonged to a musical group and formed the sample for this study. As seen in Table 1, the most common music activity was singing (*n* = 172), followed by instrumental (*n* = 48) and dance groups (*n* = 37). Participants were adults aged 17–88 years (*M* = 45.93, SD = 17.44), and the majority were female (78%). There were no significant differences in the gender breakdown for the three music activities. Participants in the singing groups were significantly older than those in the instrumental and dance groups. Half of the participants resided in Australia, with the other half residing in either the United States or United Kingdom. At the time of completing the survey, most participants were restricted to only leaving their home for essentials (72%) for the last 7–9 weeks (48%). In terms of expertise levels, approximately half of singing and dance group participants were novices, with the remaining being intermediates. Sixty-three percent of instrumental groups were at the intermediate level, with the remainder being novices. For all three music activities, approximately 10% of participants were experts.

**TABLE 1:** Demographic and COVID-19 social distancing status of the survey participants.

Variable	Full sample	Instrumental	Singing	Dance
*N*	257	48	172	37
Age *M* (SD)	45.93 (17.44)	37.98 (17.88)	50.07 (15.68)	36.78 (18.08)
Range	17–88	18–72	17–88	17–76
Female	197 (78%)	27 (59%)	137 (80%)	33 (89%)
**Residence**				
Australia	127 (51%)	29 (61%)	72 (42%)	26 (74%)
United States	66 (26%)	4 (9%)	56 (33%)	6 (17%)
United Kingdom	52 (21%)	13 (30%)	36 (21%)	3 (9%)
Other	6 (2%)	0 (0%)	6 (4%)	0 (0%)
**Social distancing circumstance**				
Self-isolating: COVID contact	0 (0%)	0 (0%)	0 (0%)	0 (0%)
Self-isolating: contact with someone exhibiting symptoms but not positive	2 (<1%)	0 (0%)	2 (1%)	0 (0%)
Leaving home only for essentials	186 (72%)	31 (65%)	128 (74%)	27 (73%)
Leaving home as essential worker	50 (20%)	15 (31%)	27 (16%)	8 (22%)
Not social distancing	1 (<1%)	0 (0%)	1 (<1%)	0 (0%)
Other	18 (7%)	2 (4%)	14 (8%)	2 (5%)
Duration of social distancing				
1–3 weeks	3 (1%)	2 (4%)	1 (<1%)	0 (0%)
4–6 weeks	61 (24%)	14 (29%)	33 (19%)	14 (38%)
7–9 weeks	122 (48%)	27 (56%)	79 (46%)	16 (43%)
9+ weeks	71 (28%)	5 (10%)	59 (34%)	7 (19%)
**Group activity expertise**				
Novice/open to all	121 (47%)	13 (27%)	88 (51%)	20 (54%)
Intermediate	114 (44%)	30 (63%)	70 (41%)	14 (38%)
Expert	22 (9%)	5 (10%)	14 (8%)	3 (8%)

### Power Analysis

To achieve adequately powered analyses, *a priori* sample sizes were calculated using previous literature effect sizes and G^∗^Power (Faul et al., 2007; Kyprianides and Easterbrook, 2020; Lonsdale and Day, 2020). The calculated sample sizes needed for all analyses were exceeded. For a one-way ANOVA of group type for Q1, a minimum total sample size of 216 participants was required, which was exceeded. Seventy-five participants whose musical group adapted virtually were required for Q3, which was exceeded.

### Measures

#### Group Activity

Participants were asked to choose one group activity that they were involved in before the onset of social distancing and to describe the nature of it through purpose-written questions about the frequency of meetings, duration of membership, the number of people in the group, and the expertise level of the group. Participants were asked to complete measures of group identification and psychological need satisfaction by retrospectively reflecting on their music group in F2F mode. Next, we asked respondents whether their music group had adapted to social distancing by meeting electronically. Those whose groups did not adapt were asked to describe (in an open text box) why they believed this to be the case. For those whose groups did adapt (*n* = 153), participants were asked questions about the nature and success of the adaptation and to complete group identification and psychological need satisfaction measures a second time in relation to this virtually adapted music group.

#### Group Identification

The Group Identification Scale developed by Doosje et al. (1995) includes four items (e.g., “I identify with others in this group” and “I see myself as a member of this group”). Responses were recorded on a 5-point response scale, from 1 (*strongly disagree*) to 5 (*strongly agree*). Scores were calculated by averaging the four items, with higher scores indicating stronger identification with a group. This measure is psychometrically acceptable (Postmes et al., 2013) and had good reliability in this sample (F2F: α = 0.874; virtual: α = 0.870).

#### Psychological Need Satisfaction

The 7-item psychological need satisfaction scale used by Kyprianides and Easterbrook (2020) was adapted to include three more items derived from social identity theory relating to support given and received by ingroup members: group-based emotional regulation 1 (“I share my feelings with others in the group”), group-based emotional regulation 2 (“I provide emotional support to others in the group”), and the opportunity to help others (“I make a contribution to the group”; Haslam et al., 2005). The original seven items explored the needs of social support (“I receive support from others in the group”), self-esteem (“being a part of the group gives me self-esteem”), competence (“being part of the group gives me a sense of achievement”), relatedness (“I am close and connected to other people in the group”), autonomy (“I can speak my mind in the group”), control (“I have control over my activities in the group”), and meaning (“being a part of the group gives my life meaning”). A 5-point response scale was used, from 1 (*strongly disagree*) to 5 (*strongly agree*). A global psychological need satisfaction score was calculated by averaging the 10 items, with higher scores indicating greater satisfaction of psychological needs. In this sample, the measure had good to excellent reliability (F2F: α = 0.896; virtual: α = 0.929).

#### Mental Health

The mental health score from the 12-item short form health survey (SF-12) (Ware et al., 1996) was used (e.g., “how much of the time during the past 4 weeks have you felt calm and peaceful?”). Scoring was performed using the method described in Ware et al. (1995). Some questions were reworded to be more appropriate to social distancing restrictions (e.g., a question asking about an individual’s ability to visit relatives was changed to talking to relatives instead). Lower scores indicate lower mental health functioning, where scores are compared with the norms developed for the scale (*M* = 50, SD = 10).

### Control Variables

Although not the focus of any research hypotheses, the following variables were measured to control for their potential relationships with the outcome variables.

#### Extraversion

The 4-item extraversion subscale from the 20-item Mini-IPIP scale was used (Donnellan et al., 2006). Responses range from 1 (*very inaccurate*) to 5 (*very accurate*) and scoring totals the responses. Higher scores indicate higher extraversion. Two items (e.g., “I don’t talk a lot”) are reverse scored, whereas the remaining two items (e.g., “I am the life of the party”) are scored normally. This is a psychometrically acceptable personality measure that had acceptable reliability in this sample (α = 0.766; Donnellan et al., 2006).

### Procedure

Recruitment used social media, where the Qualtrics survey was advertised in public Facebook groups for choirs, orchestras, and social sports. A snowballing method was used, where participants invited their friends and group members to complete the survey. Participants were also recruited through the UQ psychology research participation scheme, where first-year students receive course credit for participating in research studies. The survey was advertised as research designed to explore whether people were missing their group activities during COVID-19, and it took about 15 min to complete. Informed consent was obtained prior to starting the survey, and participation was voluntary. Participants could elect to go into a draw to win one of five $100 (AU) online shopping vouchers or equivalent in their country’s currency. Approval for the study was obtained by both the UQ and Goldsmiths University ethics committees.

## Results

### Preliminary Data Checks

There were no significant differences between the UQ and Goldsmiths data sets on most participant characteristic values presented in Table 1 so data were merged together. Although the two data sets were not comparable on location, social distancing circumstance, and duration, this was expected as the UQ version sampled mainly Australians and the Goldsmith version sampled mainly United Kingdom and United States participants who were impacted more severely by COVID-19. Missing data were assessed, and 17% of data were deleted on the basis that these participants missed more than 50% of data because they withdrew from the survey before completing. Little’s MCAR test showed that data missing in the remaining cases were missing at random. Expectation-maximization algorithm was used to impute missing data. F2F group identification was negatively skewed that was expected due to participants self-selecting into a study about “missing your group.” The untransformed version of this variable produced the same result as the transformed version; therefore, the untransformed data were presented for ease of interpretation. The potential relationships between the control variables and the independent and dependent variables were assessed. The only significant correlations observed were between social distancing duration and both mental health [*r*(237) = −0.27, *p* = 0.001] and virtual group identification [*r*(151) = 0.18, *p* = 0.023]. Therefore, social distancing duration was controlled for in all analyses involving mental health and virtual group identification.

### Group Identification and Psychological Need Satisfaction in Various Music Groups

The first question explores if any differences exist across group music activities relating to how members identified with their F2F groups and satisfied their psychological needs prior to COVID-19. To explore this, data from the whole sample in relation to their F2F music group activities were used in a one-way (musical group type: instrumental, singing, and dance) between-groups ANOVA (*n* = 244) on group identification. There was no significant main effect of musical group type on group identification, *F*(2, 241) = 1.86, *p* = 0.158, η_*p*_^2^ = 0.02, indicating that group identification was similar among instrumental (*M* = 4.55, SD = 0.48), singing (*M* = 4.50, SD = 0.48), and dance groups (*M* = 4.35, SD = 0.53).

**FIGURE 1:**
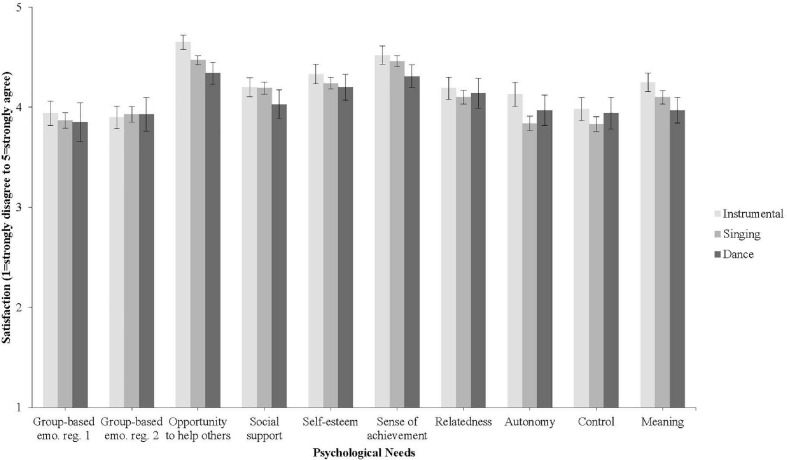
Mean ratings of satisfaction of ten psychological needs in instrumental, singing, and dance group (retrospectively rated for face to face groups pre-COVID-19). Differences in ratings between music groups were not significant. Bars are standard errors.

Next, a series of one-way between-groups ANOVAs was performed to determine if there was any difference between music groups in psychological need satisfaction. Tests of homogeneity were not met for two needs–opportunity to help others and need for control–therefore a more conservative significance level (α = 0.025) was adapted for these items. Interestingly, as Figure 1 shows, there were no significant differences between music group types in satisfaction of any of the 10 psychological needs: sharing feelings in the group, *F*(2, 157) = 0.010, *p* = 0.990, providing emotional support to others in the group, *F*(2, 157) = 0.169, *p* = 0.844, opportunity to help others, *F*(2, 157) = 2.806, *p* = 0.064, receive social support from others in the group, *F*(2, 157) = 0.583, *p* = 0.560, self-esteem, *F*(2, 157) = 0.149, *p* = 0.862, sense of achievement, *F*(2, 157) = 0.192, *p* = 0.825, relatedness, *F*(2, 157) = 0.809, *p* = 0.447, autonomy, *F*(2, 157) = 2.352, *p* = 0.099, control, *F*(2, 157) = 0.672, *p* = 0.512, and meaning, *F*(2, 157) = 0.502, *p* = 0.606.

The second question was whether there was a difference between music activity groups in the rate of adaptation to virtual forms during COVID-19. A Pearson’s chi-square test of independence revealed a significant difference between adaptation rates for the three groups, χ^2^(2, *N* = 253) = 14.13, *p* = 0.001, *V* = 0.24. Follow-up *Z* tests for proportions were conducted, where instrumental groups (59.6%) had a significantly lower adaptation rate than singing (83.4%) and dance groups (86.5%), *Z* = −3.50, *p* < 0.001, *h* = 0.54 and *Z* = −2.71, *p* = 0.007, *h* = 0.63, respectively. Singing and dance groups were not significantly different, *Z* = −0.47, *p* = 0.642, *h* = 0.09.

Participants were given an open text box to describe why their musical group did not adapt, and representative responses from instrumental group members are included in Table 2. Some of the reasons that prevented groups from adapting included group members lacking technological expertise, an inability to find appropriate technology, difficulty organizing and coordinating a large group (e.g., orchestras with 90 members), lacking funding and other resources to facilitate the transition to an electronic platform, or unwillingness to adapt as virtual modes lack the same F2F “energy.”

**TABLE 2:** Selected open text responses about why instrumental groups did not adapt to virtual mode.

No, because we are a musical group. It is hard to synchronize our singing and playing through the internet as it often lags.
It was held at a venue that had to close for COVID reasons, but the hope is to re-open when restrictions ease.
It is not very easy to coordinate a group as large as this online/over video chat. This is because sometimes the video/sound lags and it is not possible to have the same energy when you’re playing through video chats.
It is hard to do music online together.
Because there’s roughly 90 people in our orchestra and getting all of us together on an electronic platform would be very difficult. Also, the orchestra is run by a non-for-profit organization that relies largely on concert ticket sales to keep running—with no concerts on at the moment, they have to use their limited resources sparingly.
Hard to play music over the internet.
Because we cannot play together online, it doesn’t work.
We don’t have each other’s phone number.
No, I cannot find good quality/cost affordable/easy audio platforms to be able to perform simultaneously. Also, older membership so knowledge of technology required would leave some members out.
Technological difficulties—we had a boardgames night over zoom, but it is impossible to hold a rehearsal over video conference when playback is at different speeds.
We have mainly talked on the phone. There are two main singers, and we arrange the harmonies. It is not the same having a singing rehearsal *via* zoom, and we already have a large repertoire. Also, the main part about rehearsals we enjoy is hearing each other’s voice in the same room.
Delays in online conferencing make this near impossible.
It was too difficult due to varying levels of technical expertise within the orchestra.

As well, for those whose groups did adapt, participants in dance and singing groups rated the adapted group as more successful than participants in instrumental groups. In terms of the nature of the adapted group, it is clear that competition played a relatively insignificant role for all groups, whereas features, such as skill practice, chatting, and catching up, played larger roles across all three music activities. In particular, chatting and catching up informed virtual adaptations for singing groups to a greater extent. There were also other activity-specific features, such as virtual singing groups were more likely to engage in making sounds without hearing others, whereas dance groups were more likely to engage in virtual forms of physical exercise or synchronizing movements together. The third question was whether people participating in the virtual adapted groups felt similar levels of group identification and, in turn, psychological need satisfaction with their adapted vs F2F music group. An ANCOVA and an ANOVA were performed using data only from participants that adapted virtually (*n* = 153). Data were collapsed across musical group types as this effect was expected to be generalizable across musical groups. Tests of homogeneity were not met for either analysis, so a more conservative alpha level (α = 0.025) was adapted. A one-way (mode: F2F and virtual) between-groups ANCOVA was performed on group identification, with social distancing duration entered as a covariate. There was a significant main effect of mode on group identification, *F*(1, 303) = 54.07, *p* < 0.001, η_*p*_^2^ = 0.15, such that group identification for the virtual group (*M* = 4.04, SD = 0.69 out of 5) was significantly lower than that for the F2F group (*M* = 4.53, SD = 0.46). Similarly, a one-way between-groups (mode: F2F and virtual) ANOVA was performed for psychological need satisfaction (averaged across the 10 items). A significant main effect of mode on psychological need satisfaction was revealed, *F*(1, 304) = 62.59, *p* < 0.001, η_*p*_^2^ = 0.17, such that virtual music groups satisfied participants’ psychological needs (*M* = 3.50, SD = 0.81 out of 5) significantly less than music groups in F2F mode (*M* = 4.14, SD = 0.56).

**FIGURE 2:**
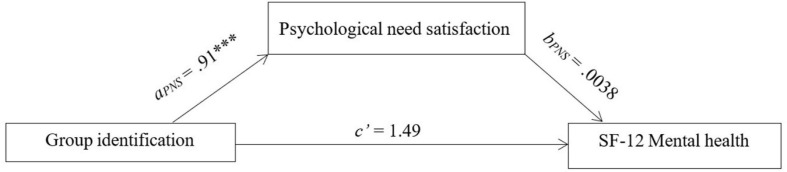
Meditation model for direct and indirect effects with associated significance level and understand regression coefficients using bootstrapping. PNS, psychological need satisfaction; ^∗∗∗^*p* < 0.001, *N* = 157.

The final question for the study was whether psychological need satisfaction would mediate the relationship between virtual music group identification and mental health. A bootstrapping analysis using Hayes (2018) PROCESS v3.3 macro was performed to assess the indirect effect. Estimates were based on 5,000 samples, and unstandardized coefficients were calculated as shown in Figure 2. Group identification and psychological need satisfaction for virtual groups were the hypothesized predictor and mediator, respectively, with SF-12 mental health as the outcome. Path *a*_*PNS*_ was significant, *a*_*PNS*_ = 0.91, *p* < 0.001, whereas the total and direct effects of group identification on mental health were not significant, *c* = 1.49, *p* = 0.259 and *c*’ = 1.49, *p* = 0.513, respectively. There was no indirect effect, indicating that average psychological need satisfaction did not mediate the relationship between group identification and mental health, *b* = 0.003, SE_*boot*_ = 1.85, 95% CI_*boot*_ = [−3.70, 3.62]. A planned moderated mediation analysis to control for social distancing duration on the relationship between group identification and mental health was not conducted due to the lack of support for the mediation model.

## Discussion

The restrictions relating to physical contact caused by the COVID-19 pandemic allowed us to examine whether musical groups adapted to a virtual format, and if so, to what extent this adapted form met participants’ psychological needs, in comparison with the F2F original form. First, the study extended earlier work involving singing groups (Livesey et al., 2012; Williams et al., 2019; Dingle et al., 2020) and the “Finding your Rhythm” music group (Kyprianides and Easterbrook, 2020) to a range of other instrumental and dance groups. We found no difference between instrumental, dance, and singing groups on mean group identification, which was rated between “agreed” and “strongly agreed” (4–5 out of 5). This may simply reflect the way the study was advertised as the “missing my groups during COVID-19” study. Clearly, this recruitment approach attracted a sample of people who identified strongly with their musical groups. The lack of differences in identification between music activity groups is in line with previous social identity research that demonstrates that the group activity does not influence identification (Cruwys et al., 2014; Williams et al., 2019), rather it is the extent to which people feel a good “fit” and sense of belonging with their chosen group.

More surprising was the finding that there was no difference between music group activities in terms of their capacity to meet specific psychological needs. As the mean values in Figure 1 show, participants “agreed” (rated four out of five on average) that all 10 psychological needs were satisfied by their music group, with negligible differences between the groups. Previous research has established that group singing meets individuals’ psychological needs for relatedness, belonging, and support (Livesey et al., 2012); both group singing and group creative writing meet participants’ needs for belonging, support, self-efficacy, purpose, and positive emotions (Williams et al., 2020); scores for autonomy were significantly higher for solo singers than for choir singers and team sport players, whereas group entitativity (relatedness) was higher for choir singers than for team sport players (Stewart and Lonsdale, 2016), and that group music making and recording meets a range of psychological needs (Kyprianides and Easterbrook, 2020). To the authors’ knowledge, however, this is the first study to establish a lack of significant difference between instrumental, singing, and dance groups in satisfying 10 psychological needs for participants. This finding suggests that, regardless of what the group does together, psychological needs flow from individuals’ identification with the group, which is consistent with social identity theorizing (Jetten et al., 2011, 2014; Haslam et al., 2018).

Turning to the question about the potential effects of COVID-19 social distancing on musical groups, the findings showed that instrumental groups (60%) were less likely than singing and dance groups (80%) to adapt to virtual modes. The open text responses shown in Table 2 suggest that because instrumental groups tended to be more at the intermediate level, whereas singing and dance groups tended to be at the novice level (as seen in Table 1), instrumental members saw less value in meeting virtually if it was not expected to work properly or if they could not perform for an audience. Furthermore, the responses demonstrate that technological requirements are not the only barrier preventing instrumental groups from adapting. This information illustrates that several financial, technological, and logistical factors need to be considered for a musical group to successfully adapt virtually. These issues were also explored in the study by Daffern et al. (2021) of choir singers and facilitators during COVID-19 lockdown. These researchers identified that “Co-creation through Singing” was important to their respondents, who described “an overwhelming sense of loss of the embodied experience of singing together in real-time, which is unattainable from existing virtual choir models” (Daffern et al., 2021, abstract).

Nevertheless, these adaptation rates were strikingly high, given that previous organizational psychology research found that individuals who are high identifiers to a group are opposed to any changes or restructuring of that group (Jetten et al., 2002). In our study, it appears that individuals (especially those in dance and singing groups) were motivated to continue contact with their music group during COVID-19, and that adapted groups engaged in other activities, such as skills practice, synchronized movements, sharing ideas, and general catching up and conversation. Individuals generally seek to restore positive social identities when they are threatened by an event (e.g., social distancing; Jetten et al., 2017), so it is possible that this motivated participants to continue groups online. Future research should investigate this further by examining group members who join virtual musical groups (such as Whitacre’s virtual choir) by choice rather than under the circumstances of a pandemic.

Group identification and average psychological need satisfaction were lower in the virtually adapted groups than in the reported experience in a F2F format. This was a large effect that is comparable to other effect sizes demonstrating this finding (Knippenberg et al., 2002). The lower rating for group identification suggests that an individual’s sense of self was being informed by the virtual group to a lesser extent compared with the F2F group and consequently, the virtual group had lower capacity to satisfy its member’s psychological needs. This may have been because in many cases, the virtual musical group was unable to play synchronously or to hear one another’s voices in harmony like they would when F2F, and this detracts from the whole group experience. Furthermore, non-musical elements, such as touch and conversation, are curtailed in virtual modes, and these features may be equally important to group members as the musical elements in producing group identification and meeting psychological needs (E. Williams et al., 2020; Dingle et al., 2020). Yet despite the lower scores for group identification and psychological need satisfaction for virtual groups compared with the F2F mode, scores were still relatively high as they were above the scale midpoints and were comparable to other values in the literature for musical groups on these outcomes (Stewart and Lonsdale, 2016). This suggests that the virtual groups are still effective at maintaining identification and psychological need satisfaction to some extent.

Finally, the results showed that psychological need satisfaction did not mediate the relationship between group identification and mental health, which is inconsistent with previous research in music groups (Kyprianides and Easterbrook, 2020; Williams et al., 2020). In fact, the mental health of participants was not related to either group identification or psychological need satisfaction, which was unexpected as group identification is known to be associated with a wide range of wellbeing and mental health outcomes (Jetten et al., 2014; Haslam et al., 2018). This may be because people’s mental health during COVID-19 was likely to have been influenced by a range of factors beyond music group identification, such as changes to work and study, loss of work, loneliness, lack of personal space in the home, increase in domestic and family violence, and increase in hazardous drinking (Holmes et al., 2020). The influence of music group participation may have been lost among all of these other determinants of mental health and wellbeing. Nevertheless, some support for the social identity approach was observed, since a significant pathway between group identification and psychological need satisfaction was found. That is, the more participants identified with the group, the greater psychological need satisfaction they experienced.

### Limitations and Implications

A limitation of the study is its cross-sectional design, which means that we are unable to determine the potential changes in virtual music groups and their effects on identification and psychological needs over time. For instance, it may be that some people’s early enthusiasm for participating in virtual music groups wanes over time, or conversely, that early difficulties with technical and practical issues are resolved over time so that the virtual group experience improves. The cross-sectional survey meant that scores were all based on self-report when many of the groups were no longer occurring, and it could be that participants find it difficult to reflect on activities they are not currently involved in. Another limitation of the study was the unequal sample sizes across the three music activity groups, although ANOVA is relatively robust to this, and we checked for homogeneity of variance and used adjusted *F* test results where this assumption was not met.

Limitations aside, it appears that an individual’s capacity to identify with their group and satisfy their psychological needs is not impacted by musical group type, and that musical groups satisfy a broad range of psychological needs. As such, a novel finding of this study is that dance and instrumental groups provide for physical and mental health in similar ways to singing groups. To determine if this finding is generalizable to other non-music groups (e.g., sewing, sport, gaming groups), future research could explore this by examining the non-music data that were also collected in our broader study. This study also demonstrated which needs are satisfied through musical groups that is valuable for musical group facilitators as they can design activities and sessions around satisfying these needs. For example, to satisfy the need for meaning, facilitators can have group members work toward a collective outcome, such as a performance, as being a part of something bigger than oneself may create a sense of meaning (E. Williams et al., 2020). To satisfy participants’ needs for belonging and relatedness, music group leaders could ensure that members are welcomed to each session, and that rehearsals include a break in the middle or after the rehearsal to promote social interactions among members (e.g., Tarrant et al., 2016; Dingle et al., 2019).

Until COVID-19 can be successfully managed, it is unclear when and to what extent musical groups will be able to return to F2F modes. This may take longer than other activities as aerosol transmission has been linked to woodwind and brass instruments and vocal loudness (K. Williams and Underhill, 2020). Fortunately, this study suggests that virtual adaptations are still beneficial for musical groups and the continuation of virtual adaptations is recommended. As well, it is likely that advances in online technology and physical barriers (e.g., separating group members into tents so they can still perform in person) can offer music groups more ways to continue engaging with their groups. For instance, researchers at the University of Birmingham are developing ARME (Augmented Reality Music Ensemble)—a virtual “timing-sensitive” rehearsal platform that can allow solo musicians to rehearse with a group of virtual colleagues based on audio-visual recordings of professional players (University of Birmingham News, 2021). Further research is needed to understand how these methods influence the experience of virtual music-making. Furthermore, compliance among members is likely to be high as groups appeared to be committed to maintaining contact. These results suggest that virtual musical groups are a viable option for individuals who are unable to physically attend meetings, such as remote residents, people with physical disabilities, or immunocompromised patients (Fancourt et al., 2019).

## Conclusion

Overall, singing, instrumental, and dance groups appear to have similar levels of group identification and psychological need satisfaction, and people’s preference is likely to be a more important determinant of identification than type of musical activity. Furthermore, this study demonstrates that group identification and psychological needs rely to some extent on musical group members meeting F2F. Despite this, virtual groups can still provide significant benefits, and most individuals appeared to be motivated to adapt and continue meeting.

## Data Availability Statement

The raw data supporting the conclusions of this article will be made available by the authors, without undue reservation.

## Ethics Statement

The studies involving human participants were reviewed and approved by Human Research Ethics Committee, The University of Queensland. The patients/participants provided their written informed consent to participate in this study.

## Author Contributions

GAD designed the study in collaboration with colleagues at Goldsmiths University of London. GD refined the specific questions for this study and led the analysis and wrote the music group data presented from the broader project. Both authors had input into the writing of the manuscript for publication.

## Conflict of Interest

The authors declare that the research was conducted in the absence of any commercial or financial relationships that could be construed as a potential conflict of interest.

## Footnotes

1 Due to slightly different ethics processes at the two universities, the two surveys required different participant information and consent information at the start. Furthermore, the UQ survey was launched a few weeks earlier than the United Kingdom survey because the first wave was coming to an end in Queensland and social activities were starting to recommence so our opportunity to collect data about people missing their groups was rapidly coming to an end. The United Kingdom authors made some further amendments to the survey to improve the clarity of some items.
